# Carbon fiber coated by quinoa cellulose nanosheet with outstanding scaled salt self-cleaning performance and purification of organic and antibiotic contaminated water

**DOI:** 10.1038/s41598-022-12889-9

**Published:** 2022-05-24

**Authors:** Jie Yang, Xidong Suo, Jingjing Zhao, Jing Wang, Runye Zhou, Yu Zhang, Yifei Zhang, Hongtao Qiao, Xiaohang Luo

**Affiliations:** Department of Chemistry, Xinzhou Teachers University, 1 Dun Qi Street, Xinzhou, 034000 Shan Xi China

**Keywords:** Energy harvesting, Renewable energy, Materials for devices

## Abstract

To date, various solar driven evaporation technologies have been developed for treatment of seawater and wastewater but with the threat from salt polluted and single treatment of seawater. Herein, we develop a multifunctional evaporator constructed by carbon fiber coated by quinoa cellulose nanosheet (CFQC) with outstanding self-cleaning performance and good purification property for treatment of organic and antibiotic polluted water. The resulting Zn-CFQC exhibits good light to thermal performance which can absorb about 86.95% lights in the range of UV–Vis–NIR (200–2500 nm); therefore, the wet and dry surface temperatures of Zn-CFQC are held at 62.1 and 124.3 °C respectively, and keep a speed of 3.2 kg m^−2^ h^−1^ for water evaporating under 1000 W m^−2^ illumination. Such good light-to-thermal capabilities can be mainly imputed to the unique surface microstructures of the carbon fiber which decorated by two-dimension cellulose and activated by ZnCl_2_. Additionally, Zn-CFQC shows good salt automatic-cleaning capability at night and corresponding mechanism has been simply elucidated according to the chemical potential theory. The method of treatment of carbon fiber opens a new way for commercial carbon fiber utilization of solar assisted water purification.

## Introduction

The challenges of energy and clean water scarcity, especially in the remote areas, are becoming a more and more serious issue and would severely influence on economic and society development^1,2^. Currently, many technologies have been proposed to solve these problems^3–5^, for instance reverse systems^6,7^, multi-stage flash^8,9^, adsorbed treatment^10^, tiny-fog collection^2,11,12^ and interface solar assisted evaporation^13,14^, among which solar assisted evaporation is regarded as a promising strategy to address the fresh water scarcity by treating the seawater on account of its economically, easy operation, renewable energy sources, sustainability and environment friendliness^15,16^. The biggest advantage of interfacial evaporation is high solar energy utilizing efficiency which attributes to its excellent energy management by remarkably suppressing heat loss to bulk water via thermal insulation foam between the bulk water and work interface, and good water management enabled by hydrophilic properties of photo-thermals conversion materials^13,17–19^. In consequence, there are a large number of scientists engaged in related field research and many kinds of photo-thermal conversion materials have been developed successfully^20,21^. However, the most reported materials suffer from disadvantages such as susceptibility to salt contamination, complex preparation processes, and difficulty in scale-up, which seriously hinder the progress of practical applications. Therefore, designing and fabricating a photothermal material with easy scale-up, salt resistance, and long-term stability and multifunctional field utilization is urgent and important for the development of solar assisted evaporation.

There are many candidate materials for solar assisted evaporation and a lot of them have great promising prospects for practical application, such as plasmonic materials^22,23^, semiconductors^24,25^, carbon-based materials^26–28^, and polymers^2,21,29,30^. Additionally, some new advanced technology has been introduced in solar steam generation field to enhance the water evaporation efficiency^31–35^, such as atomic layer deposition (ALD)^31^ technology, Janus materials fabricating technology^30,32,33,36^, piezoelectric and solar steam synergistic technology^35,37^ and photovoltaic and solar steam generation synergistic technology^23,38^. For photo-thermal conversion materials, carbon materials have attracted largely interests in virtue of its excellent chemical stability, thermal stability, broadband absorption of solar lights, and extensive sources from nature and industrial goods^13,39–41^. Carbon fiber (CF) as commercial products with the performance of “light and strong” is widely used in advanced composites materials (aerospace, military, sports, automobile and others applications) due to its excellent properties, including low density, outstanding mechanical properties, corrosion resistance, creep resistance, chemical stability, good thermal conductivity, and peculiarly good adsorption of sunlight^42–45^. In spite of so many merits of CF, its hydrophilicity of surface is too poor of hydrophilicity to be directly utilized for solar assisted evaporation owing to absence of polar functional groups^46^. Many efforts have been made to improve the hydrophilic ability of carbon fiber for water vapor evaporation, including treatment of nitric acid^47^, plasma treatments^48^, hydrothermal method^46^, coated by graphene^49^. Although these technologies have good results, the material manufacturing methods are too complex and expensive to be widely utilized. Therefore, it is significant to exploit new method for improving the hydrophilicity of CF and utilization of solar assisted water purification.

Herein, we first develop a multifunctional evaporator which manufactured by carbon fiber and quinoa bran cellulose nanosheet (QBC). Various activated agents, including KOH, H_3_PO_4_, CuCl_2_ and ZnCl_2_, are used to treat the carbon fiber and cellulose composites, in order to make differences in their surface microstructure and improve their solar evaporation performance. Besides evaporating seawater, the evaporator based on Zn-CFQC also exhibits good capability in the treatment of organic and antibiotic contaminated water. Moreover, Zn-CFQC also exhibits a great salt self-cleaning performance for 3.5 wt% and 7.0 wt% saline water. Here, we exploit a convenient technology to purify the seawater, organic and antibiotic polluted water for production fresh water by utilizing commercial carbon fiber and agriculture by-product.

## Results and discussion

### Quinoa cellulose nanosheet coated carbon fiber composites

**Figure 1:**
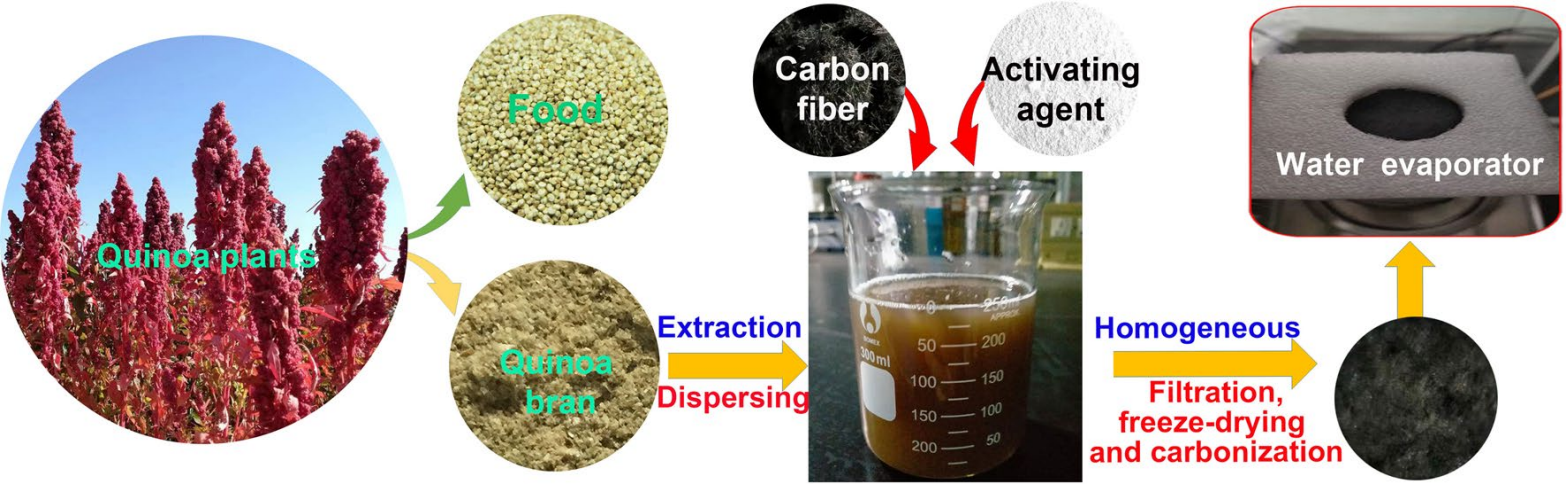
Schematic exhibition for the fabrication steps of CFQC based evaporators. Farmland site is located in Jingle County, Xinzhou City, Shanxi Province, China. Quinoa plants is photographed by Jie Yang, Department of Chemistry of Xinzhou Teachers University.

Figure 1 illustrates the manufacturing process of the CFQC which was made from CF and QBC with the assistance of vacuum filtration, cryodesiccation and high temperature carbonization processes. The quinoa bran were accumulated and washed by purified water, and then successively treated by benzene/absolute ethanol composite solution, 10 wt% NaClO_2_ (pH 4 ~ 5) and 2 wt% KOH for discarding pectin, lignin, hemicellulose and other chemicals^50^. Resulting product was dispersed in purified water by alternately treating with ultrasonic and homogenizer, and then mixed with activating agent (10 wt%, KOH, H_3_PO_4_, CuCl_2_ and ZnCl_2_) and CF (50 wt%) using a ultrasonic cleaner for obtaining the homogeneous compound dispersion. Finally, CFQC based evaporators were harvested with the quinoa cellulose nanosheet coated carbon fiber after vacuum filtration, freeze drying and pyrolyzed at 800 °C processing methods. Activation mechanism of KOH, H_3_PO_4_, CuCl_2_ and ZnCl_2_ are summarized in supplementary information. The prepared evaporators were labeled as CFQC, K-CFQC, P-CFQC, Cu-CFQC and Zn-CFQC for representing no activating agent, and activated by KOH, H_3_PO_4_, CuCl_2_ and ZnCl_2_, respectively.

**Figure 2:**
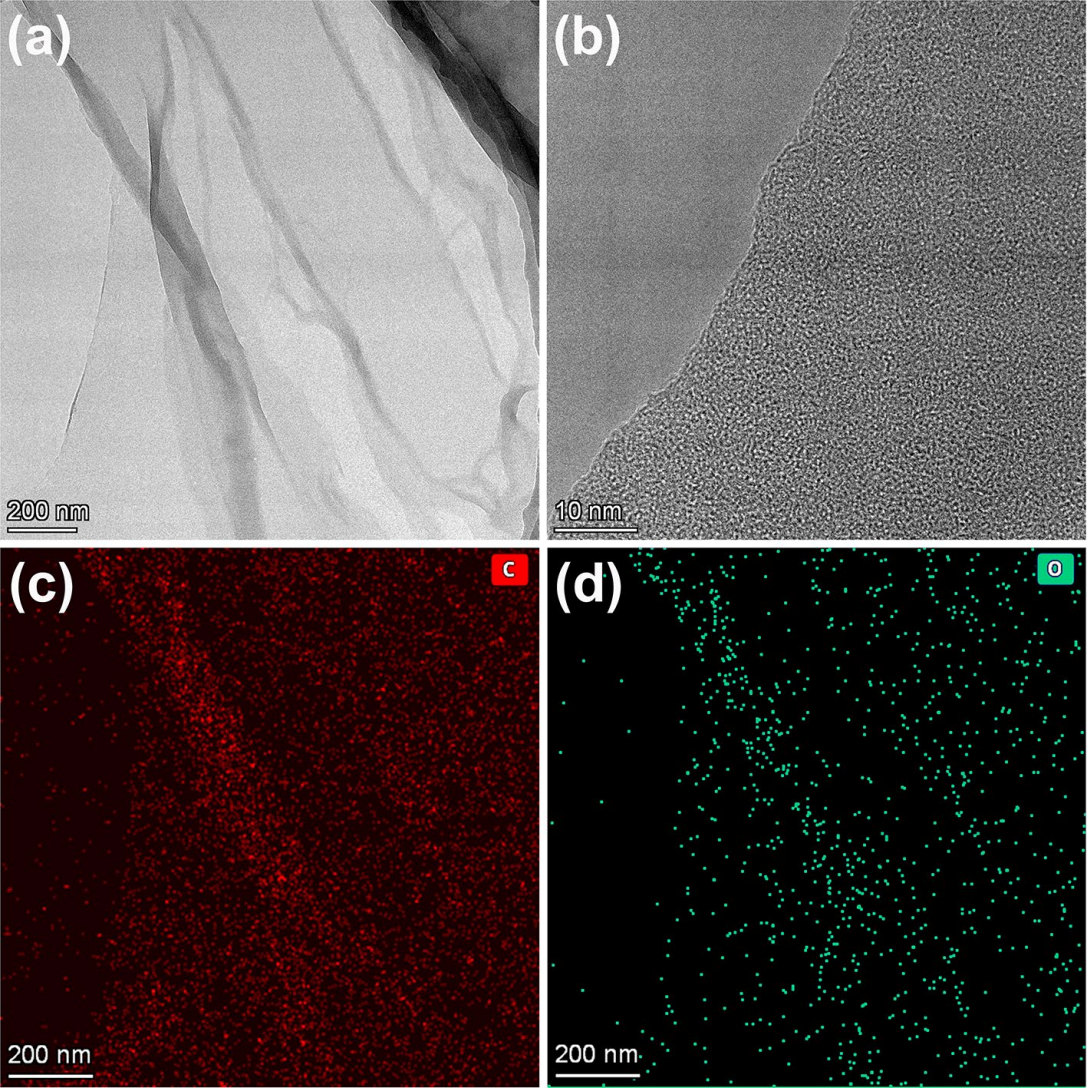
(**a**) and (**b**) TEM image of quinoa cellulose nanosheet, (**c**) and (**d**) EDS mapping images of the quinoa cellulose nanosheet.

Chemical composition and crystalline structure of the QBC has been investigated and analyzed by XRD and FTIR in our previous work^41^, and corresponding results as shown in Fig. S1. The previous results show that we successfully prepared type I cellulose from quinoa bran. For further investigating the microstructure, we employed the transmission electron microscope (TEM) to verify microscopic morphology of the QBC. As shown in Figs. 2a and b and S2, QBC interestingly exhibits two dimensional (2D) large flakes with the size beyond of 7 μm × 1.5 μm and amorphous structure. Meanwhile, the elemental mappings were determined by EDS, and corresponding results shows in as Fig. 2c and d. It is easy to find that QBC nano sheets are composed by carbon and oxygen elements, which is consistent with the FTIR results (Fig. S1).

**Figure 3:**
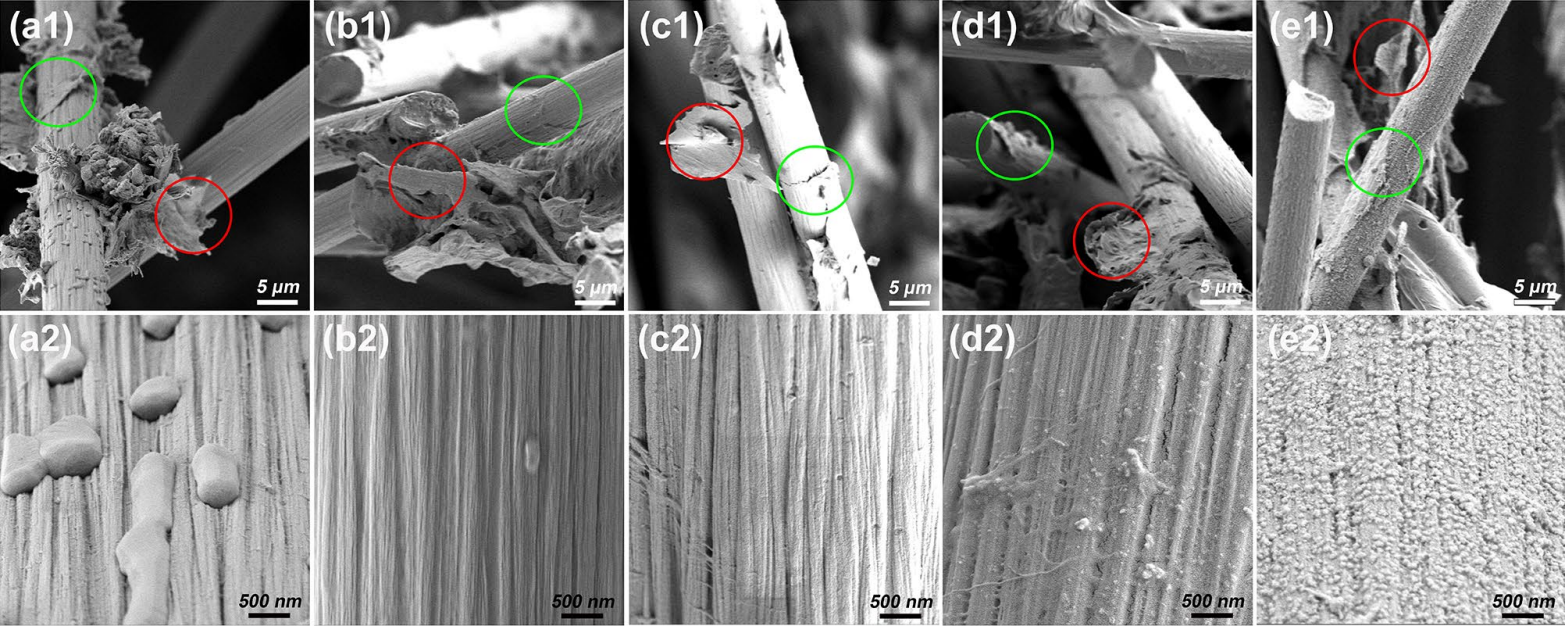
The SEM photographs of the cross sections and surface morphologies of CFQC based composite evaporators. Sample: (**a**) CFQC, (**b**), K-CFQC, (**c**) P-CFQC, (**d**) Cu-CFQC and (**e**) Zn-CFQC, (1) and (2) for cross sections and carbon fiber surface morphologies respectively.

The morphologies of all CFQC-based evaporators were surveyed with the help of electron microscopy, and the corresponding photographs are illustrated in Figs. 3 and S3. All CFQC-based films including no activated by chemical agent sample (CFQC) exhibit typical and similar carbonized quinoa cellulose nanosheet coated carbon fiber (green circle in Fig. 3) and carbonized cellulose nanosheet (red circle in Fig. 3) coexistence structures, which indicates that carbonized quinoa cellulose nano-sheet and carbon fiber can be well combined together by covalent bond after pyrolysis at 800 °C, which were observed in previous papers^51,52^. More importantly, compared with the pristine CF (Fig. S4), surface morphologies of CFQC based samples became obviously different and rougher after coated by QBC and pyrolyzed treatment, and the surfaces were completely covered by many micro-grooves. For Zn-CFQC, the surface is significantly covered by many small particles and get more rougher, while other samples only contain micro-grooves. Such complicated surface would be favorable to the light absorption and practical evaporation applications.

**Figure 4:**
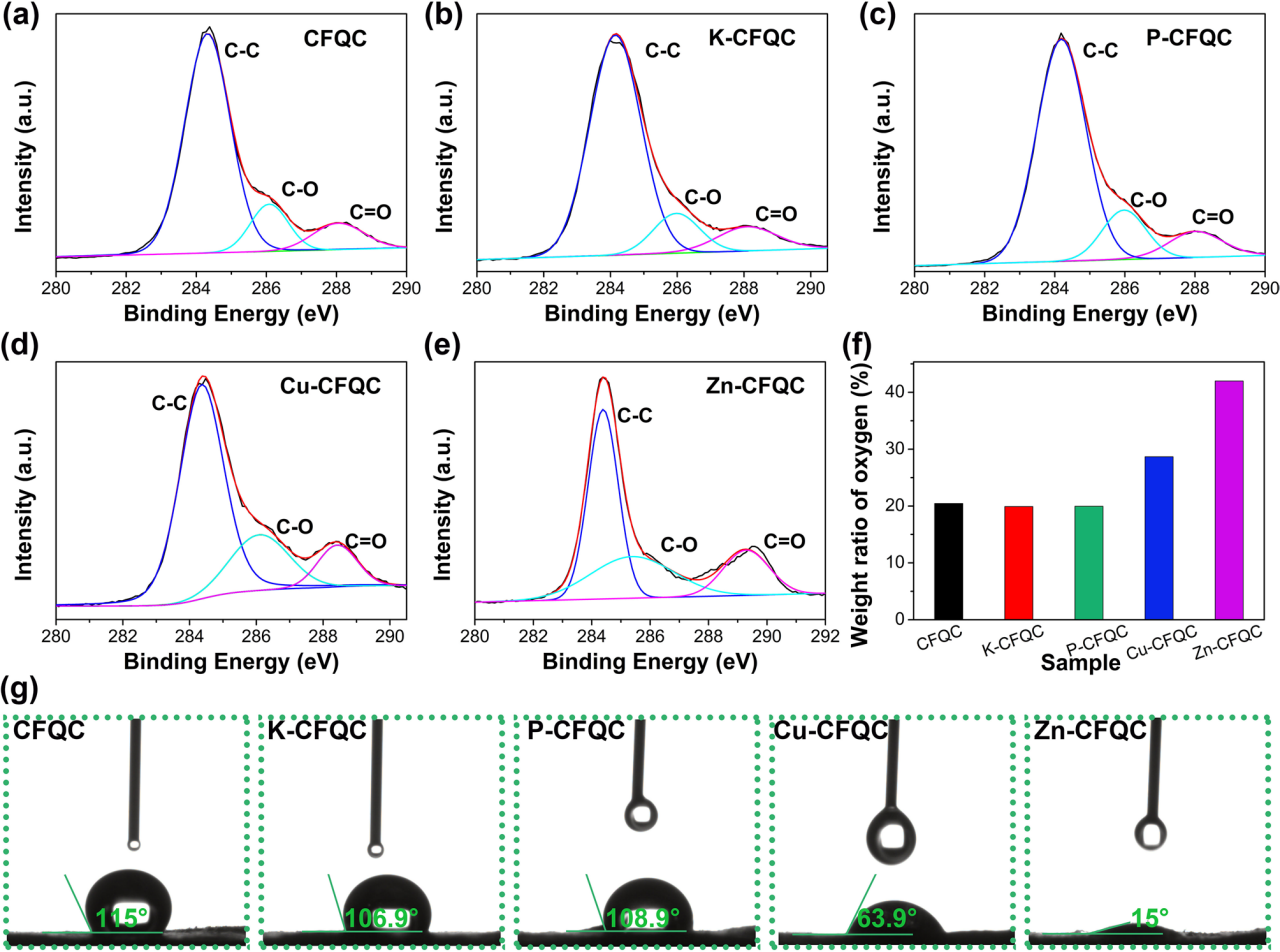
(**a**–**e**) XPS curve fitting of C 1*s* peak for CFQC, K-CFQC, P-CFQC, Cu-CFQC and Zn-CFQC. (**f**) Oxygen weight content of CFQC, K-CFQC, P-CFQC, Cu-CFQC and Zn-CFQC. (**g**) The water contact angles of CFQC based evaporators.

Additionally, functional groups of CFQC based evaporators were monitored using X-ray photoelectron spectroscopy (XPS) spectra as shown in Fig. 4a–f. From curve-fitting of C 1*s* spectra, there is clear evidence that various CFQC evaporators contains a lot of functional groups containing oxygen (C–O and C=O), which may remarkably improve hydrophilic ability by anchoring polar functional groups on carbon fiber surface. As shown in Fig. 4f, the oxygen contents of CFQC, K-CFQC and P-CFQC are around 20 wt%; whereas, the oxygen content increases from 20 wt% to 28 and 42 wt% respectively for CuCl_2_ and ZnCl_2_ activated samples. These results indicate that the samples after activated by selected metal chloride would have a high oxygen content. As shown in Fig. S5, compared to CFQC, K-CFQC and P-CFQC, water absorbing capacity of CFQC samples are significantly increased after activated by metal chloride. For directly obtaining the hydrophilic capacity of the samples, contact angle data were carefully tested and summarized in Fig. 4g. The droplets for CFQC, K-CFQC and P-CFQC are maintained at 115°, 106.9° and 108.9° respectively after 125 ms; whereas, the droplets for Cu-CFQC and Zn-CFQC are kept at 63.9° and 15° respectively after 125 ms. Contact angle data indicate that CFQC activated by metal chloride, especially zinc chloride, exhibits good hydrophilic capability, which is well consistent with the water absorbency results. Combined with the XPS data, such good hydrophilic ability for Zn-CFQC is probably due to the high content of oxygen functional group on Zn-CFQC surface. Overall, different surface oxygen content will lead to various hydrophilic ability for CFQC based evaporators. Here, we successfully exploit an efficiency method to enrich the surface functional groups and hydrophilicity of carbon fiber by mixing with unique nano-sheet quinoa cellulose and chemical activation.

### Photo to thermal conversion properties

**Figure 5:**
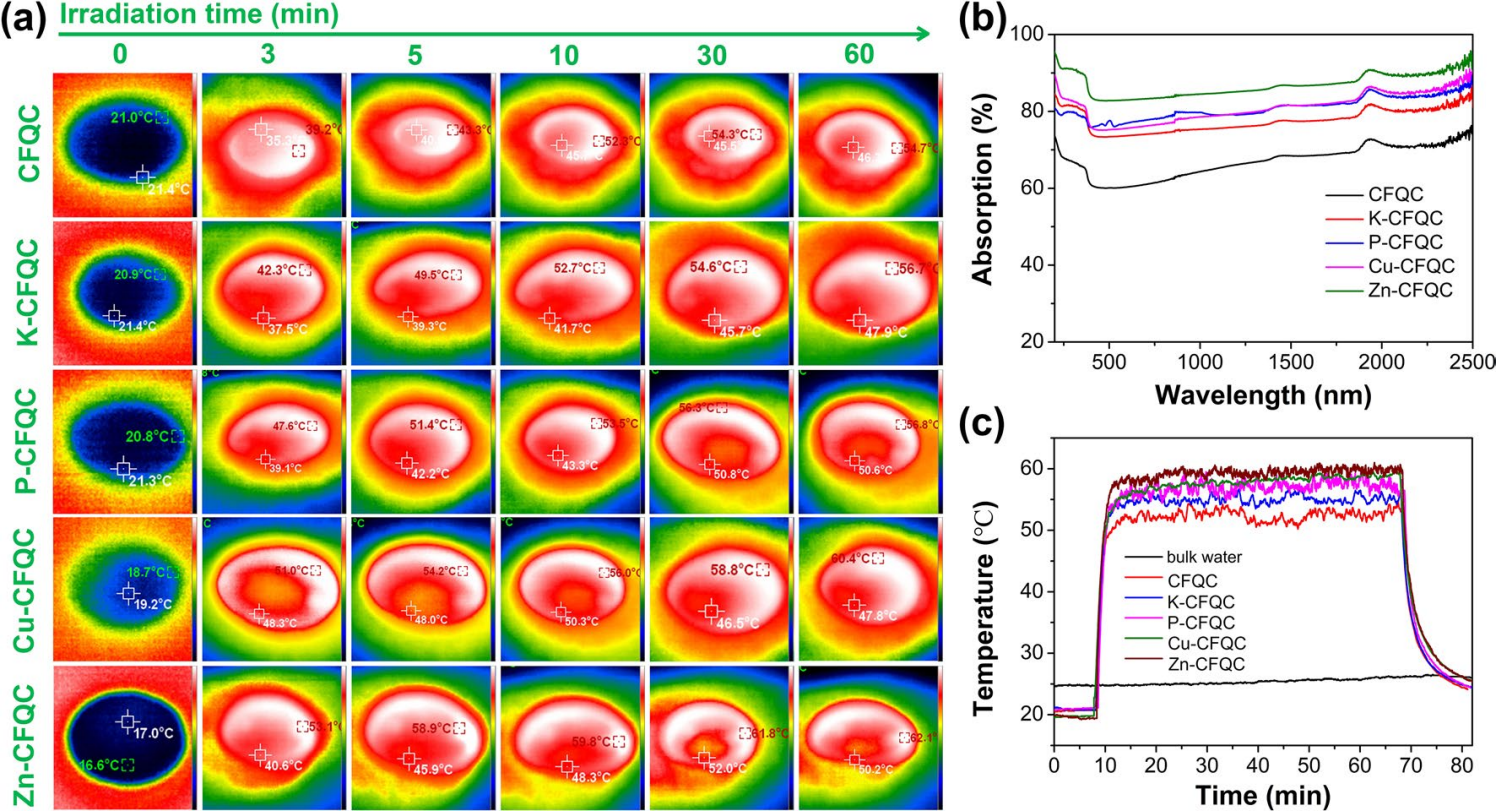
(**a**) IR camera pictures for the surface temperatures of the CFQC based evaporators during evaporation under one sun irradiation (Xenon lamp). (**b**) UV–vis–NIR absorption profiles of CFQC based evaporators; (**c**) Relative time and temperature of the wet condition of evaporators according to thermocouple under one sun (xenon lamp).

Sunlight efficient utilization of the light absorbed materials is important to solar evaporation application. To investigate the photothermal capability of the CFQC based evaporators, IR camera and thermocouple were used to measure the wet and dry surface temperature of the condition at one sun illumination under the surrounding temperature of 25 °C. Generally, the faster surface temperature of the evaporator rises and the higher the final temperature, the better light absorption and photothermal performance of the evaporator. As illustrated in Fig. 5a, the wet surface temperature of Zn-CFQC rises from 17.0 to 58.9 °C after 5 min illumination, and further increase to 59.8 °C for another 5 min, and then gradually increase to 62.1 °C, which is bigger than that of CFQC, K-CFQC, P-CFQC, Cu-CFQC for 7.4 °C, 5.4 °C, 5.3 °C, and 1.7 °C respectively. These trends also observed by thermocouple as exhibited in Fig. 5c. Additionally, the dry surface temperatures were also monitored by thermocouple to further investigate the photothermal performance. As exhibited in Fig. S6, the dry surface temperature of Zn-CFQC increase from 24.1 to 94.3 °C by 1 min illumination, and then increase to 113.4 °C by another 1 min illumination, and further ramped to 124.2 °C by another 3 min, which is higher than that of CFQC, K-CFQC, P-CFQC, Cu-CFQC for 13.5 °C, 8.6 °C, 2.0 °C, and 3.5 °C respectively. According to the temperature change trends in wet and dry condition, it is very clear that Zn-CFQC has the best photothermal conversion performance among the various CFQC based evaporators, which is consistent with the surface roughness result (Fig. 3). For better elucidating the observed photothermal performance, we carried out UV–Vis–NIR to explore light absorption abilities of CFQC based evaporators. As shown in Fig. 5b, after activated by KOH, H_3_PO_4_, CuCl_2_ and ZnCl_2_, the light absorbed performance distinctly augments from 67.05% to 77.68%, 81.57%, 80.70%, and 86.95% respectively in the range of UV–Vis–NIR (200–2500 nm). Light absorption results suggest that CFQC activated by ZnCl_2_ has stronger light absorption ability than other evaporators, which would be a directly evidence of good light to thermal performance for Zn-CFQC evaporator. Overall, the good photothermal conversion ability of Zn-CFQC is may due to the synergistic effect of its strong light absorption ability and unique surface rough structure.

### Solar water evaporation performance

**Figure 6:**
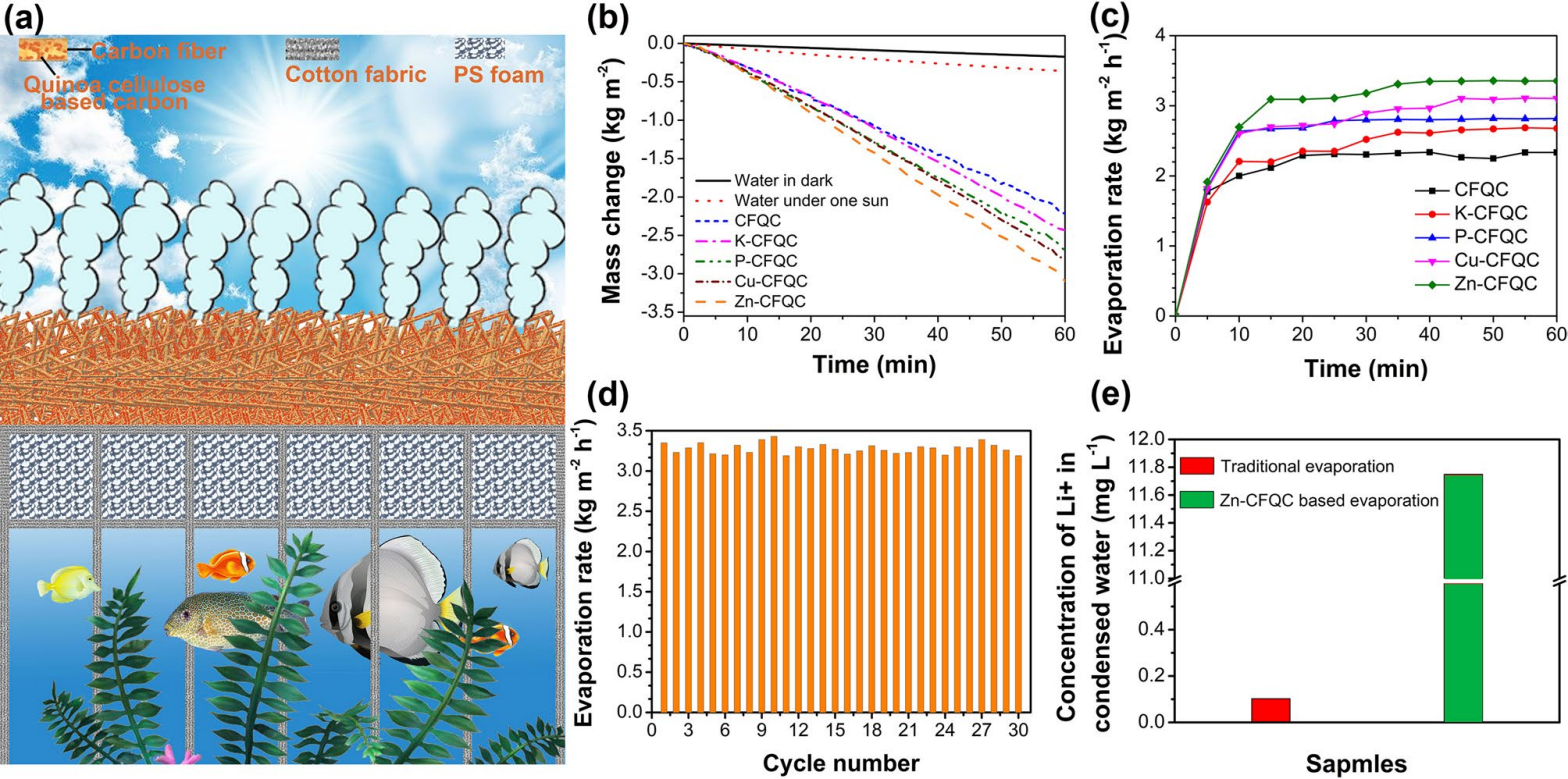
(**a**) Schematic illustration for the solar evaporation measurement system; (**b**) Water mass change versus time for CFQC based evaporators under one sun; (**c**) Time versus evaporation rate of CFQC based evaporators; (**d**) Durability of the Zn-CFQC evaporator; (**e**) Li^+^ content in condensed water collected with and without Zn-CFQC evaporator. Figure (**a**) was drawn by Jie Yang.

To systematically study the solar evaporation performance of CFQC-based materials, a home-built equipment was designed and applied to quantitatively investigate the evaporation performance of CFQC-based evaporators, where ambient conditions were kept at about 25 °C and 40% humidity. Here, a computer and electronic balance were linked and employed to record mass changes of water in vacuum container every 30 s for 1 h. As shown in Fig. 6a, the evaporator was closely attached to the cotton fabric which wrapping a PS foam to restrict the heat transference to the bulk water, where cotton fabric sagged and dipped in the bulk water to supply enough water for evaporator. The changes of water mass and water evaporation rate with time in various CFQC evaporators were illustrated in Fig. 6b and c, where light intensity was kept at one sun (1000 W m^−2^). The decrement of mass weight for pure water is only 0.17 and 0.36 kg in the condition of dark and one sun illumination, respectively; delightfully, the CFQC based evaporators exhibit distinctly high solar evaporation capability. For example, water weight reduces 2.22 kg for 1 m^2^ CFQC during 1 h illumination and its evaporation speed ramps to 2.33 kg m^−2^ h^−1^ (Fig. 6c), which is over 5.47 times greater than that on only water existence. After CFQC evaporator treated by chemical agents, water evaporated weight further rises to 2.43 kg, 2.68 kg, 2.81 kg and 3.09 kg for 1 m^2^ sample and linked evaporation speed of 2.67, 2.81, 3.10 and 3.35 kg m^−2^ h^−1^ (Fig. 6c) respectively for K-CFQC, P-CFQC, Cu-CFQC and Zn-CFQC evaporator, which is 6.75, 7.4, 7.8 and 8.5 times of that of only pure water. Figure 6d shows that the evaporation rate of Zn-CFQC based evaporator remain at 3.19 and 3.43 in the period of 30 cycles (1 h for every working cycle), which indicate that the Zn-CFQC evaporator has good durable performance for distillation application.

It should be noted that evaporation rates of CFQC based evaporator are far beyond the theoretical value under the light intensity of 1000 W m^−2^ (1.592 kg m^−2^ h^−1^, the detailed calculation process as shown in supplementary information), with apparent energy utilization rate over 100%, and those results seems unreasonable. But similar experimental phenomena have been observed in some previous literatures, such as polyvinyl alcohol (PVA) based evaporators^19,53,54^, self-blacking cellulose and graphene oxide composite gel^41^ and hollow carbon fiber^13^ have the higher evaporation rate than that of theoretical under one sun, where a water clusters based evaporation have been proposed and can be well elucidate the phenomenon of beyond the biggest value^53–55^.

According to water clusters vaporization theory, water is absorbed by some materials would have more probability to evaporate as water clusters, which would reduce the enthalpy of evaporation and require less energy than free water evaporation. To determine the form of water evaporation in Zn-CFQC, we evaporated 80 g L^−1^ LiCl solution with and without the help of Zn-CFQC under 1 sun, and accumulated two kinds of condensed water. As exhibited in Fig. 6e, the condensed water without help of Zn-CFQC contains Li^+^ of 0.1 mg in one liter, however, the Li^+^ concentration in condensed water with the help of Zn-CFQC reaches up to 11.75 mg L^−1^. The remarkable difference of Li^+^ in two different condensed water confirms that the water evaporates by the way of small clusters when Zn-CFQC existing and implies water evaporation would need less energy than that of single molecule by the way of reducing the evaporation enthalpy of water absorbed in Zn-CFQC materials according to previous studies^19,55^.

### Applications for seawater and organic polluted water

**Figure 7:**
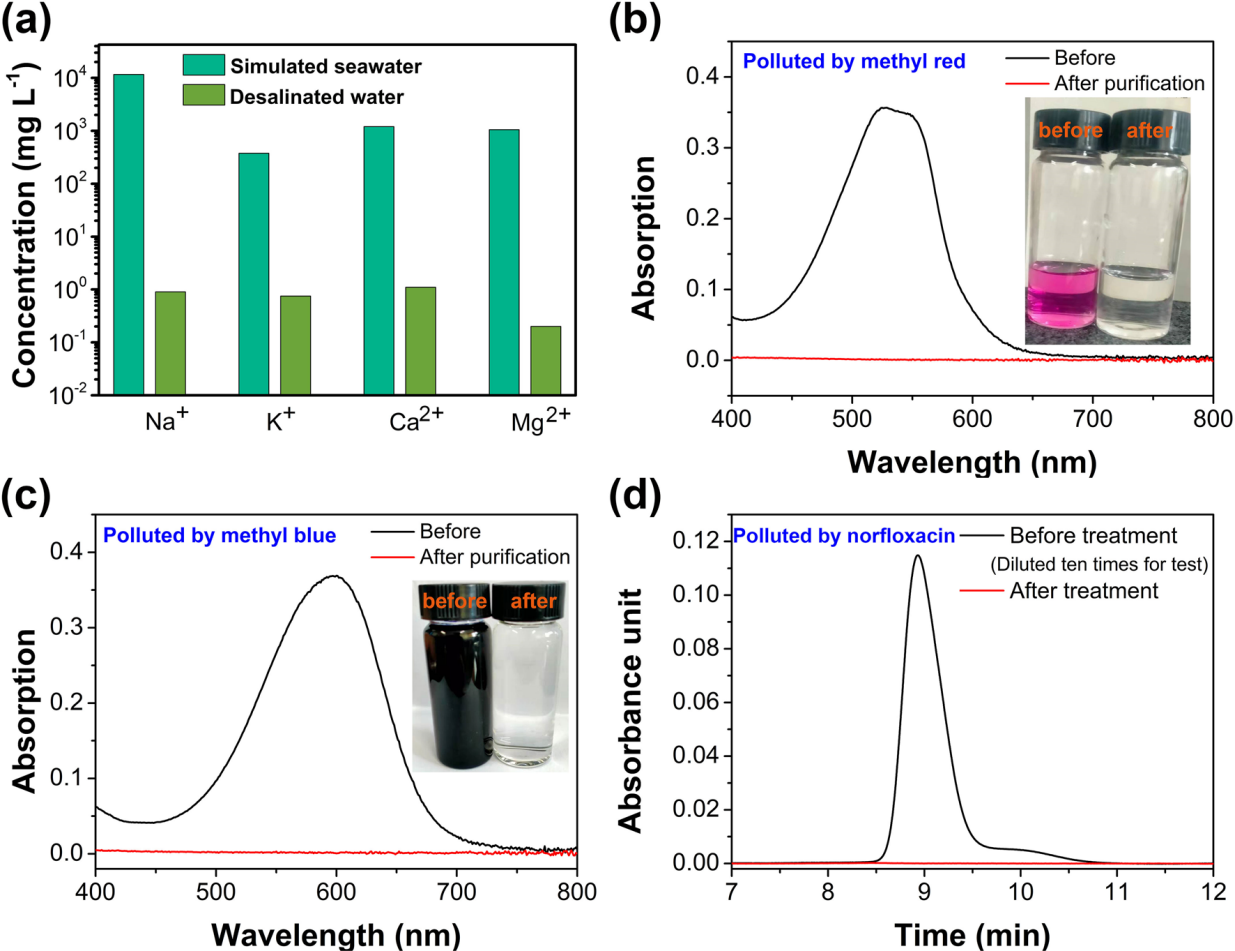
(**a**) Desalination performance of Zn-CFQC; (**b**) UV−Vis spectra and photograph of methyl red contaminated water and purified water by Zn-CFQC; (**c**) UV−Vis spectra and photograph of methyl blue contaminated water and purified water by Zn-CFQC; (**d**) HPLC curve for norfloxacin polluted water and purified water by Zn-CFQC.

The practical applications of Zn-CFQC for solar driven desalination and organic polluted water treatment were further investigated. Here, we first used the simulated seawater to evaluate the desalination performance. As illustrated in Fig. 7a, the content of Na^+^, K^+^, Ca^2+^, and Mg^2+^ of the simulated seawater are reduced from the original concentration 11,619, 376.6, 1203.2, 1052.8 mg L^−1^ to 0.9, 0.75, 1.10, 0.20 mg L^−1^ with the assistance of Zn-CFQC evaporator respectively, which indicates that seawater evaporated by Zn-CFQC can reach the standards of the World Health Organization and the Environmental Protection Agency^56,57^. Also, methyl red and methyl blue polluted water were chosen as typical organic polluted samples. As shown in Fig. 7b and c, the color of the 10 wt% methyl red and methyl blue solutions are pink and dark blue respectively; whereas, both colors of the contaminated water solutions are transformed into transparent and colorless after purification by Zn-CFQC and the strong absorption characteristic peaks of methyl red and methyl blue at about 536 nm and 597 nm are completely disappeared respectively. The disappearance of absorption peaks of methyl red and methyl blue in treated water proves that Zn-CFQC based evaporation system has the ability to purify the organic contaminated water.

Unfortunately, some freshwater rivers and lakes have been contaminated by various antibiotics, such as norfloxacin, ofloxacin, erythromycin, roerythromycin, enfloxacin, and sulfamazine^58,59^. Antibiotic pollution in lakes and rivers have become a global public concern due to their negative impact on the ecological environment and human health, especially about the antibiotic resistance problems^60^. Here, norfloxacin polluted water (0.25 g L^−1^) were chosen as a typical antibiotic polluted simulated water. As illustrated in Fig. 7d, the strong absorption characteristic peaks of norfloxacin solution at about 9 min in HPLC curve are completely disappeared after treatment by Zn-CFQC based evaporator. These results suggest that Zn-CFQC has the potential ability to purify the antibiotic contaminated water.

Above results exhibit that Zn-CFQC have good performance to purify the seawater, organic polluted water and antibiotic contaminated water, which open a new avenue to fabricate high-performance evaporator using agricultural by-products (quinoa bran) and carbon fiber.

### Self-salt cleaning performance

**Figure 8:**
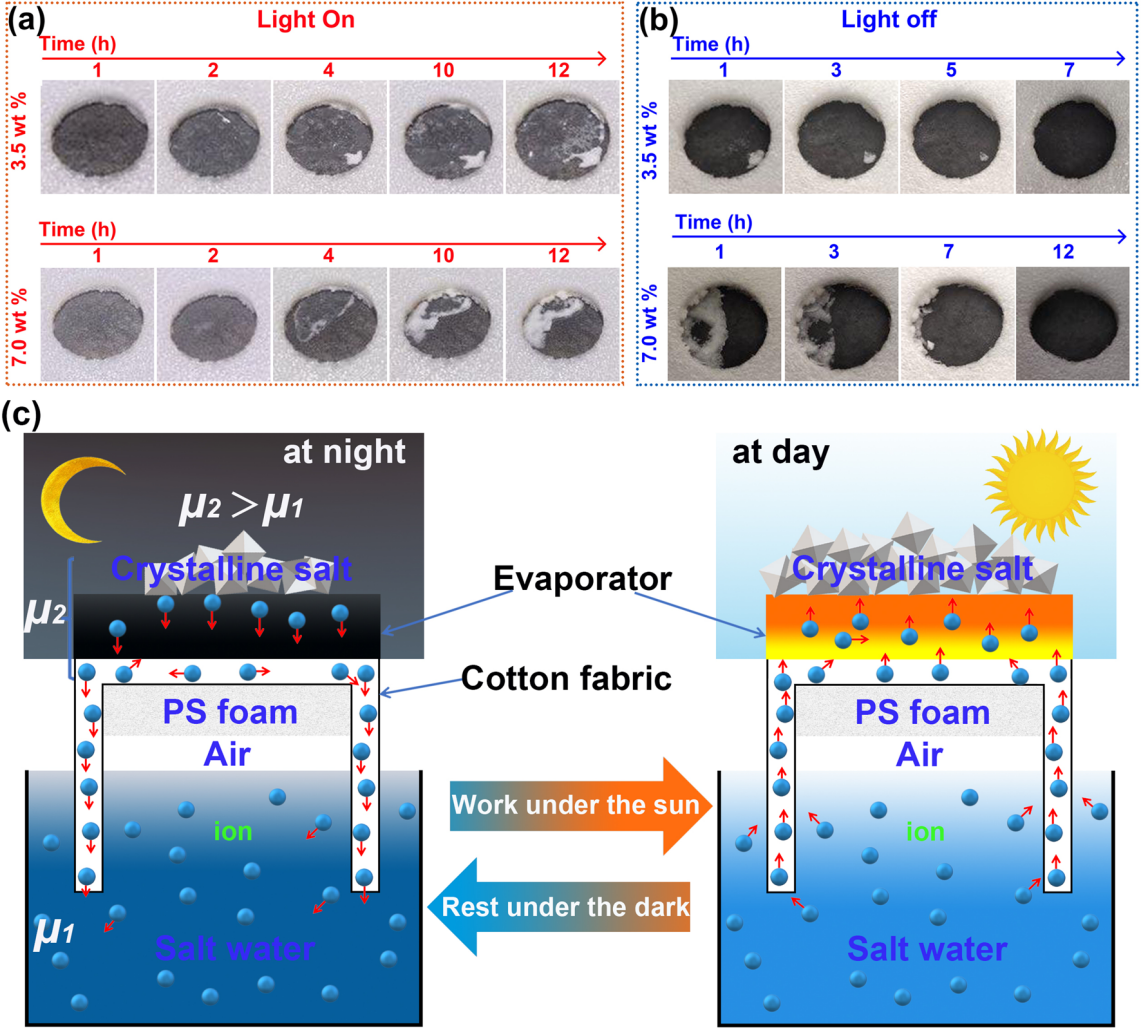
(**a**) Crystalline salt accumulation phenomena of the Zn-CFQC evaporator at laboratory under one sun; (**b**) Crystalline salt self-cleaned phenomenon at the Zn-CFQC surface during the dark; (**c**) Schematic diagram of the salt accumulation and self-cleaning mechanism of Zn-CFQC during the working time and break time.

Anti-salt performance is very important to practical application of solar evaporation evaporator because of surface accumulated salt largely diminishing the performance of the evaporator^61^. The materials possessed self-salt cleaning property need to have super-hydrophilicity and enough water transmission channel to satisfy its rapid self-salt cleaning performance, which have been reported by pervious studies^61–63^. Interestingly, Zn-CFQC shows a valuable automatic-salt-cleaning capability. As shown in Fig. 8a and b, large amounts of crystalline salt emerge on the surface of the Zn-CFQC after working for 12 h for 3.5 wt% and 7.0 wt% NaCl solution, and after a period of rest (under dark or LED light, where the light intensity of the LED was neglected) for 7 h and 12 h respectively, the surfaces salts are automatically dissolved. The results show that the salt-scale is self-cleaned from Zn-CFQC evaporator surface under the non or weak light irradiation conditions. As exhibited in Fig. 8c, the salt is slowly amassed at the surface of Zn-CFQC under one sun illumination, and then gradually returned back to the bulk salt-water during the non-working period. This phenomenon can be well explained depend on chemical potential theory. When salt accumulated on the surface, surface salt concentration is higher than that of bulk water. Chemical potential can be calculated according to the follow formulas:

1 $$ \mu_{1} = \mu^{*} \left( T \right) + RT\ln x_{1} $$

2 $$ \mu_{2} = \mu^{*} \left( T \right) + RT\ln x_{2} $$

3 $${\mu }_{2}-{\mu }_{1}=\Delta \mu =RT\mathrm{ln}\frac{{x}_{2}}{{x}_{1}}>0$$

where ${\mu }_{1}$ and ${\mu }_{2}$ is the bulk water and surface chemical potential respectively, ${x}_{1}$ and ${x}_{2}$ is the salt concentration at bulk water and evaporator surface, $\Delta \mu$ is chemical potential difference between the surface and bulk salt water.

According to chemical potential theory, surface chemical potential of salt is higher than that of bulk water. Driving force ($\Delta \mu$) between the evaporator and bulk salt water would prompt the salt to migrate back to bulk water with the help of the channels (gaps) between the decorated carbon fibers. According to the previous studies^41,62^, the marvelous salt-cleaning capability can be chalked up to adequate salt-water transmission network (gaps), which provided by 2D cellulose-based carbon decorated carbon fibers (Fig. 3) in Zn-CFQC, and good hydrophilicity (Fig. 4g). Abundant water transmitting channels and good water absorbing capacity would help salt scale to return back to bulk simulated seawater during the break time and is a precious performance for long-term solar desalination applications.

## Conclusions

By and large, we successfully exploit a solar evaporation system consisted by QB cellulose and CF with excellent stable solar evaporation performance under one sun. The resulting Zn-CFQC evaporation system can absorb 86.95% lights in the region of UV–Vis–NIR (200 ~ 2500 nm), which result in the wet and dry evaporator surface temperature reaches to 62.1 and 124.3 °C under one sun, respectively, with corresponding evaporation speed of 3.2 kg m^−2^ h^−1^. Good lights absorption and light-to-thermal capabilities can be mainly ascribed to unique surface microstructures of the carbon fiber which decorated by two-dimension cellulose and activated by ZnCl_2_. Interestingly, Zn-CFQC has the ability to purify the muti-polluted water, such as seawater, organic polluted water and antibiotic contaminated water. Also, Zn-CFQC has great value in the salt scale cleaning ability at night owing to its super-hydrophilicity and enough water transmission channel between the carbon fiber and carbon fiber and the corresponding mechanism is illustrated according to chemical potential theory. The method of treatment of carbon fiber provides a new and green way for efficient solar-driven production freshwater by treatment of seawater, organic and antibiotic contaminated water.

## Experimental section

### Materials and chemicals

Quinoa bran was collected from a local company (Huaqing Quinoa, China). Carbon fiber was kindly supplied by Zhongfu Shenying Carbon Fiber Co., Ltd. Carbon fiber were cut by scissors to be about 2 mm length. Benzene, absolute ethanol, NaClO_2_, NaOH, HCl, KOH, H_3_PO_4_, CuCl_2_ and ZnCl_2_ were got from Aladdin (Shanghai, China).

### Fabrication process of the nano cellulose sheets

Manufacture process of nano-cellulose sheets conditions is based on previous studies^50,64^ and our previous study^41^. The mainly steps are as following: (1) A benzene/absolute ethanol with the 2:1 volume ratio was applied to treat the quinoa bran (QB) with the assistance of Soxhlet extractor at the temperature of 90 °C for 360 min. (2) The resulting product after dried in an oven was immersed in 10 wt% NaClO_2_ with the pH of 4.5 at 75 °C for 5 h. (3) The dried sample were further treated by 2% NaOH at the temperature of 90 °C for 120 min, and then cleaned by distilled water and immersed in 1% HCl at the temperature of 80 °C for 120 min. (4) Target product was obtained after filtrating, washing and dried in a 60 °C oven.

### Manufacture steps of CFQC based evaporator

Ultrasonic cleaner was employed to get QBC aqueous solution with a mass fraction of 0.5% by treated for 10 h, and then added 0.5 g CF and 0.2 g activated agent in resulting solution to get homo-disperse suspensions by further treating for 1 h. 60 mL above suspension was vacuum filtrated by a nylon membrane (0.45 μm) to obtain a QBC and CF composite films. Finally, CFQC based evaporators were obtained by freeze-dried at − 60 °C for 48 h, and pyrolyzed at 800 ℃ under the nitrogen atmosphere for 1 h.

### Characterizations

The FTIR of the QBC were obtained using a Bruker FTIR spectrophotometer (TENSORII, German). The XRD data were obtained on a XRD equipment (Bruker, model D8 Discover with GADDS, German). The cross-section of the samples was surveyed by a Zeiss Sigma 300 SEM (German) with the help of gold sputter coating. The microstructure of the QBC was observed by a high resolution scanning TEM (Titan G2 60-300, America). Light absorption performance of the CFQC based evaporator were evaluated by a UV–Vis Spectrometer (Lambda1050, Germany). A contact angle meter was applied to estimate the hydrophilic capability of the samples. The contents of K^+^, Na^+^, Ca^2+^, Mg^2+^ and Li^+^ in water were tested using an ICP equipment (Agilent 5110, America). The concentrations of methyl blue contaminated and methyl red were tested by a UV–Vis Spectrophotometer (UV-2550, Shimadzu, Japan). The concentrations of norfloxacin polluted water were measured using a high performance liquid chromatograph (Waters 600, America).

### Solar evaporation experiment

A PLS-SXE300 Xenon lamp (China) were employed to evaluated the evaporation performance of CFQC based evaporators with assistance of AM 1.5 filter, where light intensity was confirmed by a FZ-A power meter (China) and water weight loss were recorded by a computer linked electronic balance. To better investigate the light-to-thermal performance, CFQC based evaporator were tightly put on a cotton cloth wrapped PS foam, where cotton cloth acted as a water pump for supplying enough water from bulk water. A ST9450 infrared camera (China) and a UT3208 thermocouple were used to follow the temperature tracks of wet and dry surface of evaporators and bulk water.

## Supplementary Information

Supplementary Information.

## Data Availability

The data of this manuscript are obtainable from the corresponding author Xidong Suo.
