# Supplementary material for: Carbon fiber coated by quinoa cellulose nanosheet with outstanding scaled salt self-cleaning performance and purification of organic and antibiotic contaminated water
Source: Sci Rep. 2022 May 24;12:8777. doi: 10.1038/s41598-022-12889-9 (PMC9130155; doi:10.1038/s41598-022-12889-9)
Supplement: Supplementary file 1 — Supplementary Information. [file 41598_2022_12889_MOESM1_ESM.docx]

***Supporting Information***

**Carbon fiber coated by quinoa cellulose nanosheet with outstanding scaled salt self-cleaning performance and purification of organic and antibiotic contaminated water**

Jie Yang, Xidong Suo^*^, Jingjing Zhao, Jing Wang, Runye Zhou, Yu Zhang, Yifei Zhang, Hongtao Qiao^*^, Xiaohang Luo

Department of Chemistry

Xinzhou Teachers University

1 Dun Qi Street, Xinzhou, Shan Xi, 034000, China

E-mail: xidsuo@126.com (X. Suo); 469494248@qq.com (H. Qiao)

**Keywords**: carbon fiber, quinoa cellulose, solar desalination, self-cleaning

**Activation mechanism of activated agents**

KOH, H_3_PO_4_, CuCl_2_ and ZnCl_2_ as conventional activated agents are used to treat the carbon fiber and cellulose composites for making differences in their surface microstructure and improve their solar evaporation performance. The activated mechanism of the KOH, H_3_PO_4_, and ZnCl_2_ are as follows^1-2^:

For KOH, it is the most effective activating agent for making activated carbon with an extremely high specific surface area. It has been universally deduced that KOH can suppress the generation of tar, lower the temperature of the activating reaction, accelerate the removal of non‑carbon constituents, and enhance the reaction rate of pyrolysis. Additionally, the development of pores during KOH activation is intrinsically associated with a gasification reaction^3^.

It is proposed that four main mechanisms contribute to the expansion of the pore network. KOH can easily dehydrate to produce potassium oxide and water at about 500 ℃ and the resulting water can react with carbon to produce CO_2_ and H_2_. CO_2_ and H_2_ would react with K_2_O to produce K_2_CO_3_ and potassium at about 800 ℃. The vapor of potassium metal (boiling point for potassium is 762 ℃) is constantly activated between the layers made of carbon atoms. The chemical equation involved in the above reaction is as follows:

2KOH K_2_O + H_2_O

C + H_2_O H_2_ + CO

CO + H_2_O CO_2_ + H_2_

K_2_O + CO_2_ K_2_CO_3_

K_2_O + H_2_ 2K + H_2_O

K_2_O + C 2K + CO

Those reactions lead to the formation of abundant micro/mesopores. Meanwhile, the produced water could serve as a physical activation agent and increase the porosity. In addition, the produced metallic K could intercalate into the carbon materials, which would expand the carbon lattices and create a highly porous structure^4^.

For H_3_PO_4_, it has two functions: ① as an acid catalyst to promote bond cleavage reactions and the formation of crosslinks via processes such as cyclization, and condensation; ② combine with organic species to form phosphate and polyphosphatc bridges that connect and crosslink biopolymer fragments. the main functions of H_3_PO_4_ are as follows^5-6^:

(1) Swell and dissolve cellulose under the 200 ℃.

(2) Accelerating the carbonization process by evenly heating in the whole materials at a relatively low temperature.

(3) Accelerating the dehydration process.

(4) Degradation and condensation of the smaller biopolymers produced as an acid catalyst.

(5) promoting bond cleavage reactions and the formation of crosslinks as follow chemical equation:

T＜450℃：Formation of phosphate esters on cellulose side chains and crosslinking


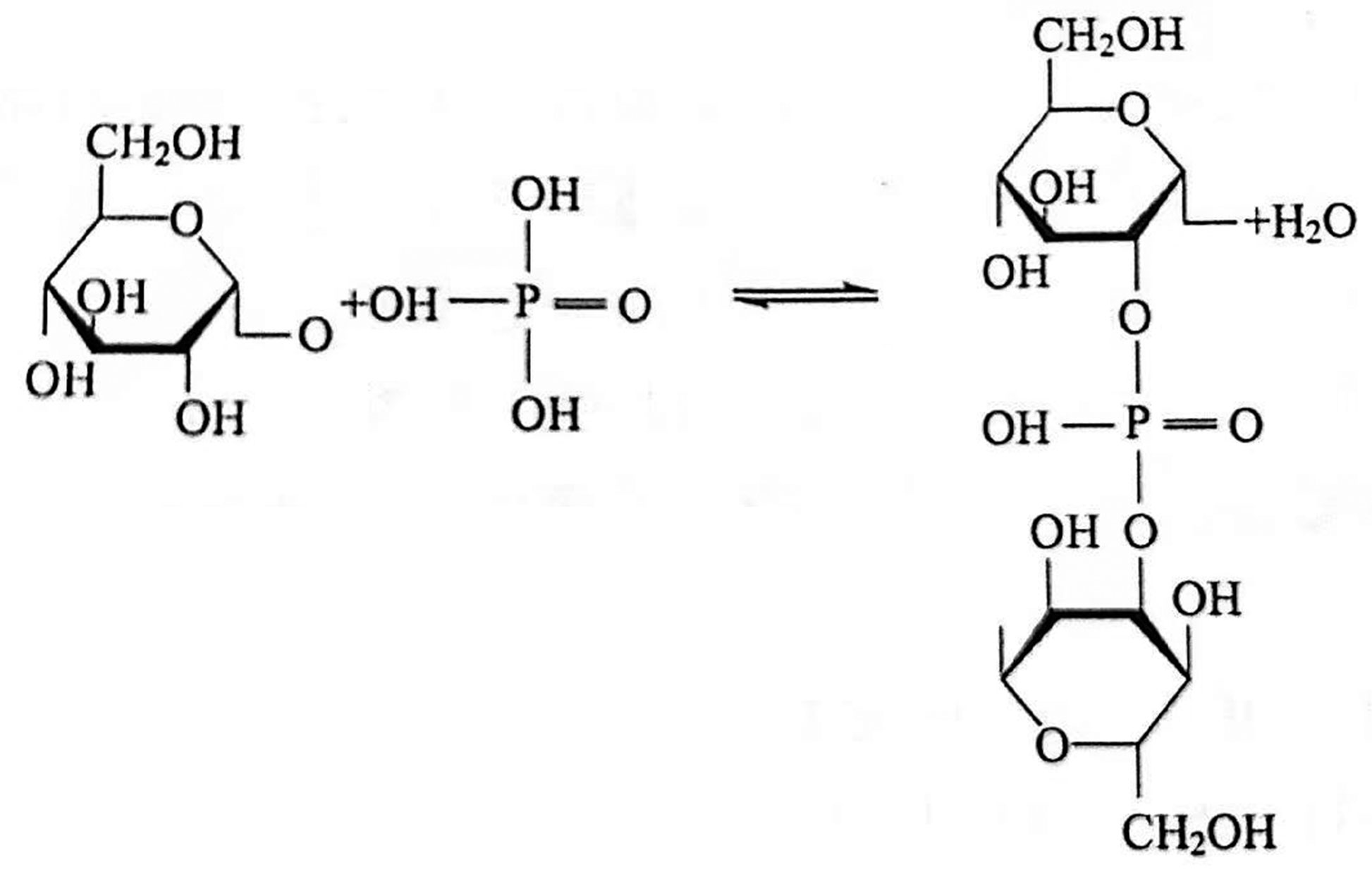


(6) Phosphoric acid would play as a framework in a carbonization process.

For ZnCl_2_, ZnCl_2_ is another important chemical activation agent for preparing porous carbon materials, and ZnCl_2_ is milder than KOH. ZnCl_2_ can catalyze the dehydration of biomass in a temperature range of 200~350 °C. The dehydration can enhance the charring and aromatization of the biomass, avoiding the formation of tar at high temperature (350~450 °C). ZnCl_2_ can increase the specific surface area of carbon materials in a temperature range of 450~600 °C. Except for the activation temperature, the amount of ZnCl_2_ is also critical to the surface properties of carbon materials^4^.

CuCl_2_ are used as chemical agents in some reports, and corresponding carbon materials exhibits good performance on the solar steam generation field^7^, but its activation mechanism is rarely studied and is not clear, which will be a focus of our next work.

**Theoretical evaporation velocity calculation**

“The theoretical evaporation speed *v* can be calculated as follows in equation:

$$v=\frac{W\times t}{h_{v}}=\frac{1000\times60\times60}{2257}=1.592Kg/m^{-2}$$

Where *W* is light power, which choose to be 1000 W m^-2^, *t* is time (1h), *h_v_* is latent heat of vaporization of water, normally taken to be 2.26 kJ g^-1^ in the region of interest^8^.


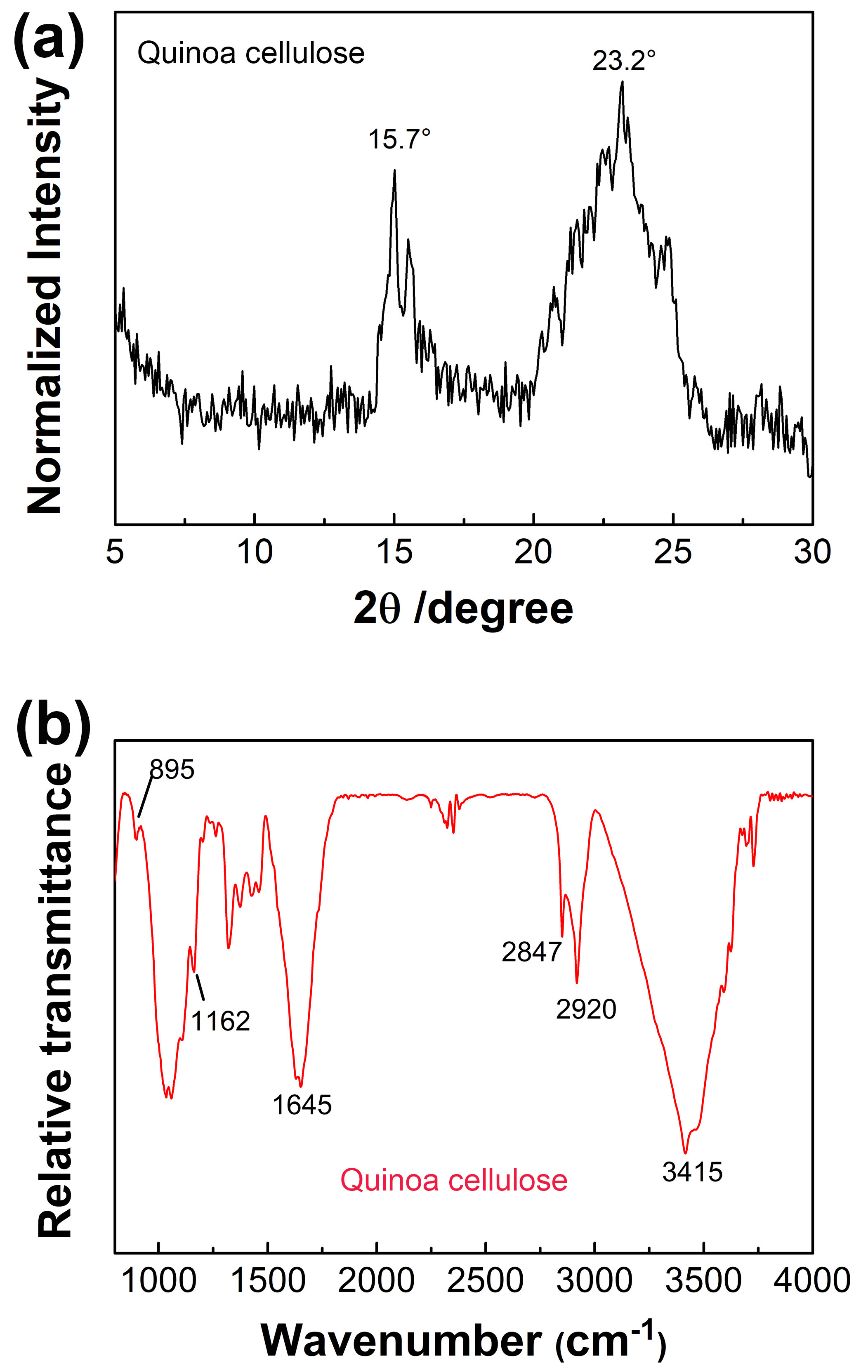


**Fig. S1.** a) FTIR spectra of the quinoa cellulose. b) XRD curves of quinoa cellulose.


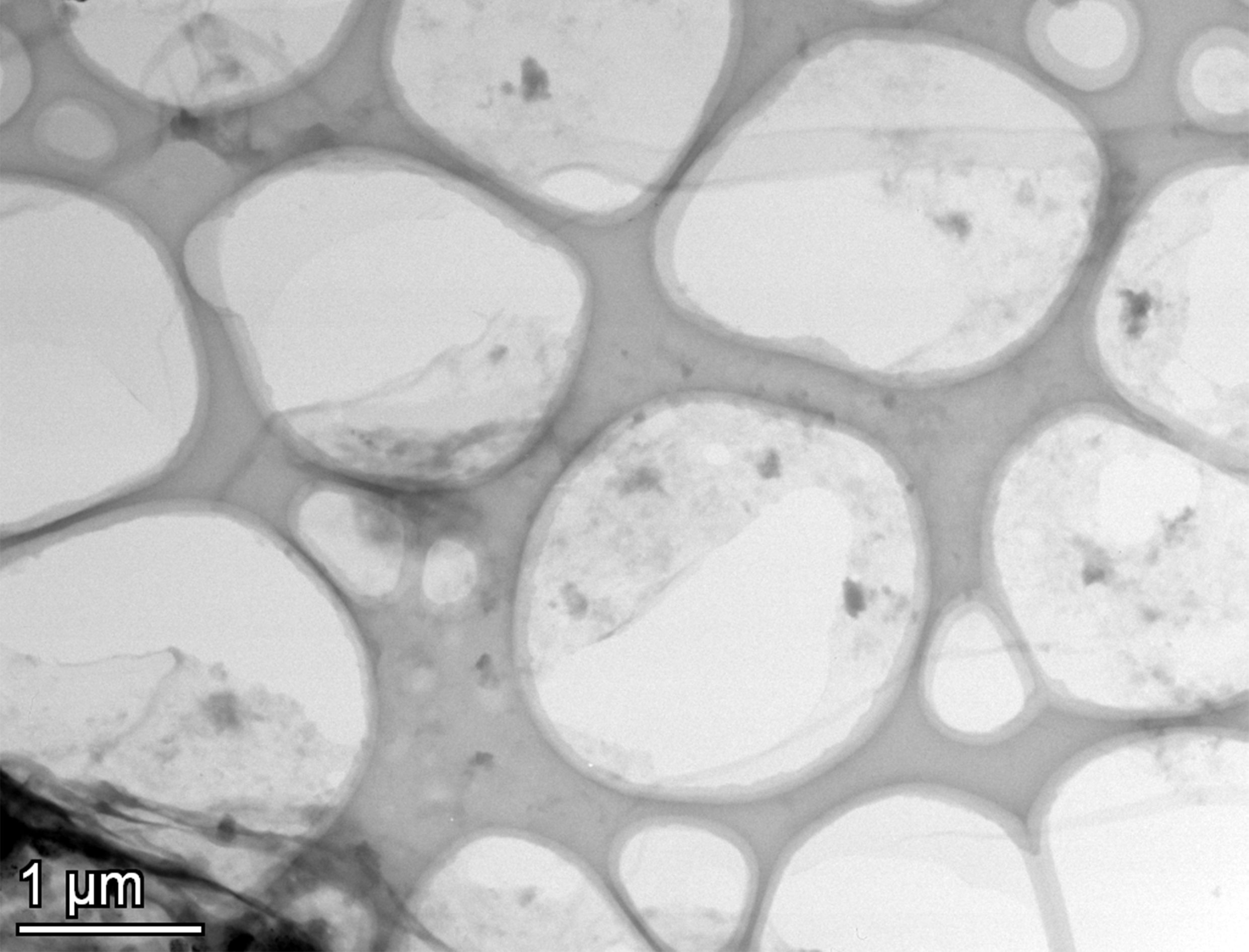


**Fig. S2.** TEM image for QBC.


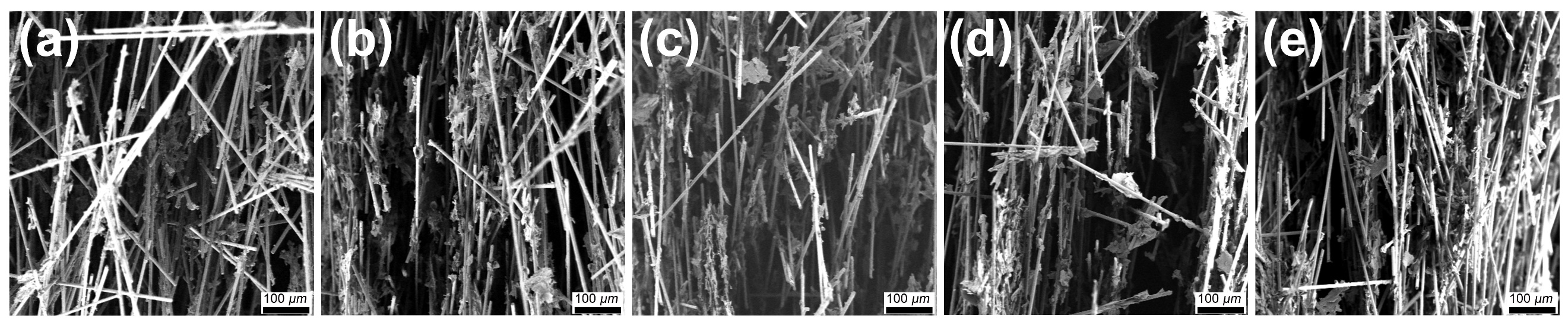


**Fig. S3** The SEM images of the cross sections of CFQC based composite carbon films. Sample: (a) CFQC, (b), K-CFQC, (c) P- CFQC, (d) Cu-CFQC and (e) Zn-CFQC.


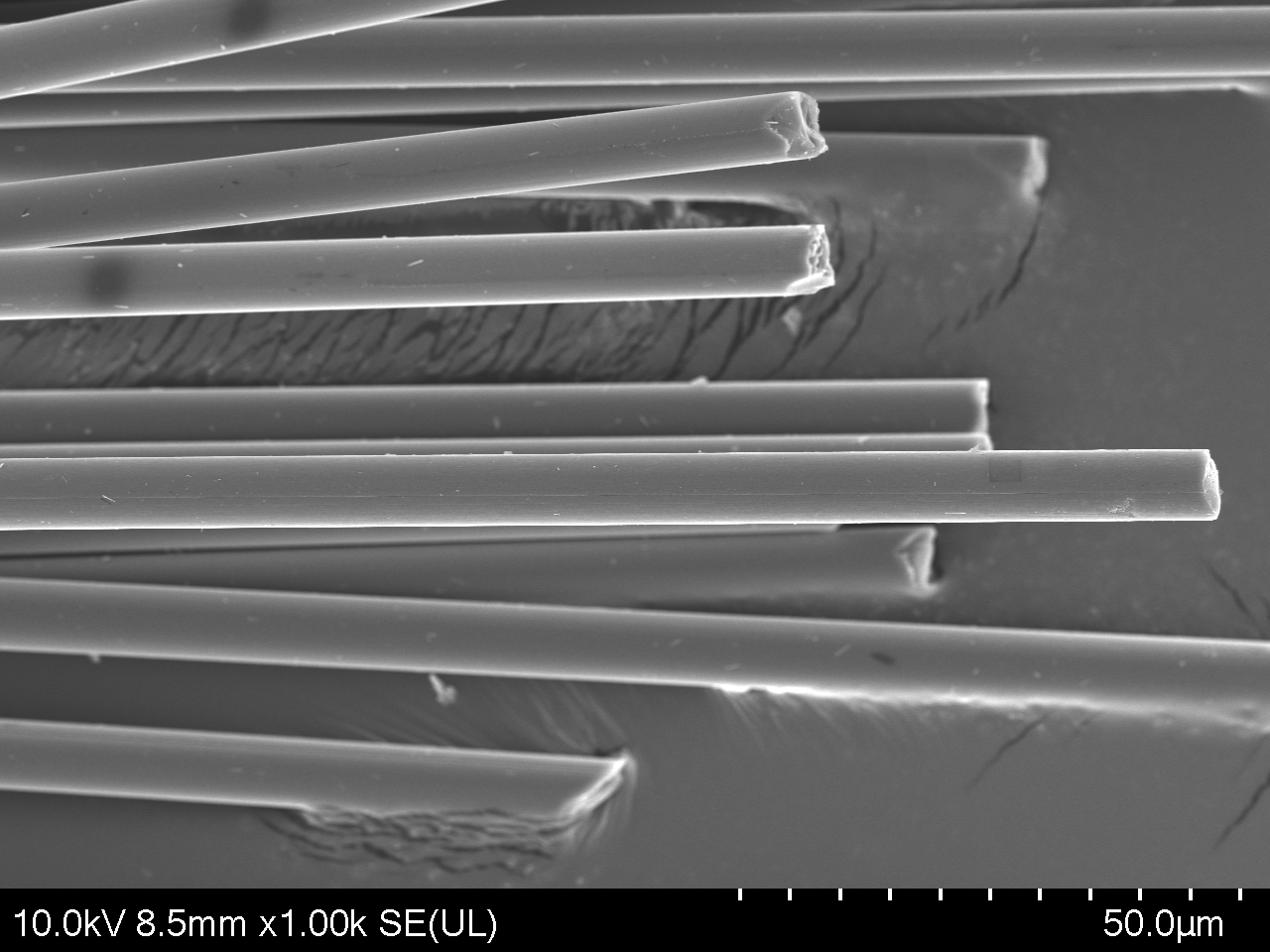


Fig. S4. Pristine carbon fiber.


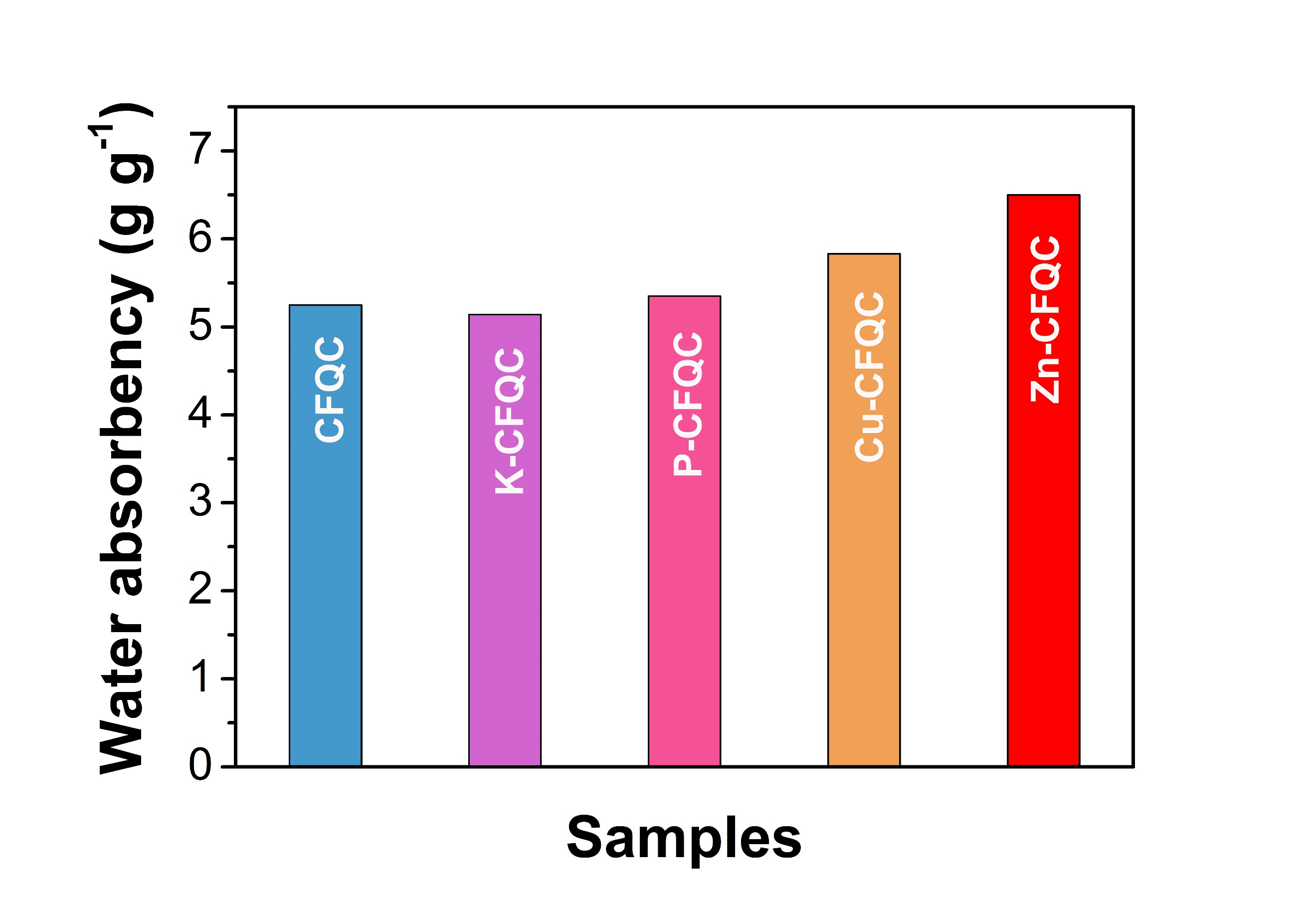


**Fig. S5.** Absorbency of the CFQC based evaporator for water.


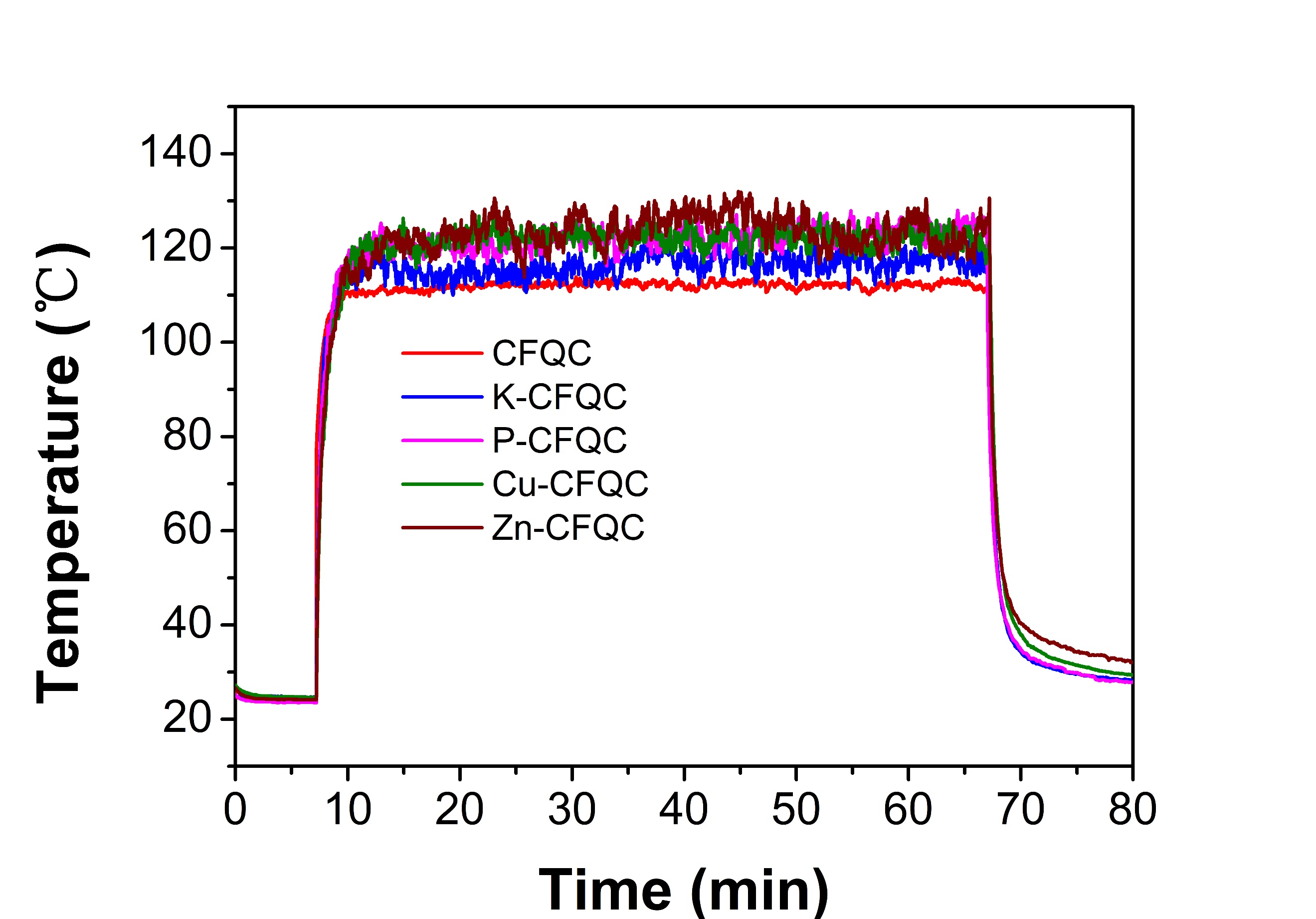


**Fig. S6.** Time-dependent temperature evolution curves of the dry surface for various CFQC based evaporators under one sun.

**References**

1. Jiang, J., *Manufacturing and application technology of activated carbon*. Chemical Industry Press Co., Lte.: China, 2017, 18-44.

2. Dolores, L.-C.; Juan, P. M.-L.; Falco, C.; Titirici, M.-M.; Diego, C.-A., Porous Biomass-Derived Carbons: Activated Carbons. *Sustainable Carbon Materials from Hydrothermal Processes* **2013**, 75-100.

3. Yahya, M. A.; Al-Qodah, Z.; Ngah, C. W. Z., Agricultural bio-waste materials as potential sustainable precursors used for activated carbon production: A review. *Renewable and Sustainable Energy Reviews* **2015,** *46*, 218-235.

4. Wang, Y.; Zhang, M.; Shen, X.; Wang, H.; Wang, H.; Xia, K.; Yin, Z.; Zhang, Y., Biomass-Derived Carbon Materials: Controllable Preparation and Versatile Applications. *Small* **2021,** *17* (40), e2008079.

5. Molina-Sabio, M.; Almansa, C.; Rodrı́guez-Reinoso, F., Phosphoric acid activated carbon discs for methane adsorption. *Carbon* **2003,** *41* (11), 2113-2119.

6. Jagtoyen, M.; Derbyshire, F., Activated carbons from yellow poplar and white oak by H3PO4 activation. *Carbon* **1998,** *36* (7), 1085-1097.

7. Tian, C.; Liu, J.; Ruan, R.; Tian, X.; Lai, X.; Xing, L.; Su, Y.; Huang, W.; Cao, Y.; Tu, J., Sandwich Photothermal Membrane with Confined Hierarchical Carbon Cells Enabling High-Efficiency Solar Steam Generation. *Small* **2020,** *16* (23), e2000573.

8. Gao, M.; Zhu, L.; Peh, C. K.; Ho, G. W., Solar absorber material and system designs for photothermal water vaporization towards clean water and energy production. *Energy & Environmental Science* **2019,** *12* (3), 841-864.
